# Forecasting the Expansion of *Bactrocera tsuneonis* (Miyake) (Diptera: Tephritidae) in China Using the MaxEnt Model

**DOI:** 10.3390/insects15060417

**Published:** 2024-06-04

**Authors:** Jianxiang Mao, Fanhua Meng, Yunzhe Song, Dongliang Li, Qinge Ji, Yongcong Hong, Jia Lin, Pumo Cai

**Affiliations:** 1 College of Tea and Food Science, Wuyi University, Wuyishan 354300, China; maojianxiang@wuyiu.edu.cn (J.M.); 202330212056@bua.edu.cn (F.M.); songyunzhe@wuyiu.edu.cn (Y.S.); 5220831073@fafu.edu.cn (D.L.); wyxyhyc@wuyiu.edu.cn (Y.H.); 2 College of Plant Science and Technology, Beijing University of Agriculture, Beijing 102206, China; 3 College of Resources and Environment, Fujian Agriculture and Forestry University, Fuzhou 350002, China; 4 Biological Control Research Institute, Fujian Agriculture and Forestry University, Fuzhou 350002, China; jiqinge@fafu.edu.cn

**Keywords:** citrus fruit fly, invasive insect, optimized MaxEnt, potential distribution, climate change, climate scenarios

## Abstract

**Simple Summary:**

*Bactrocera tsuneonis* (Miyake) is a significant pest of commercial citrus crops. It is a univoltine and oligophagous species widely distributed in Japan and China. In this study, the potential distribution of the *B. tsuneonis* population under current and different future climate change scenarios was modeled using MaxEnt software (v. 3.4.1) and optimized using R software (v. 4.3.2). Under current climate conditions, the potentially suitable areas were mainly concentrated in Central, South, and East China. The total area of habitats suitable for this pest was predicted to increase in the future climate scenarios. The centroid of the total suitable habitat for this pest gradually shifted westward and northward. Our findings provide new insights that could aid the monitoring of *B. tsuneonis* in China.

**Abstract:**

The invasive pest, *Bactrocera tsuneonis* (Miyake), has become a significant threat to China’s citrus industry. Predicting the area of potentially suitable habitats for *B. tsuneonis* is essential for optimizing pest control strategies that mitigate its impact on the citrus industry. Here, existing distribution data for *B. tsuneonis*, as well as current climate data and projections for four future periods (2021–2040, 2041–2060, 2061–2080, and 2081–2100) from the Coupled Model Intercomparison Project Phase 6 (CMIP6) were obtained. The distribution of *B. tsuneonis* under current and different climate change scenarios in China was predicted using the optimized MaxEnt model, ArcGIS, and the ENMeval data package. Model accuracy was assessed using ROC curves, and the primary environmental factors influencing the distribution of the pest were identified based on the percent contribution. When the regularization multiplier (RM) was set to 1.5 and the feature combination (FC) was set to LQH, a model with lower complexity was obtained. Under these parameter settings, the mean training AUC was 0.9916, and the mean testing AUC was 0.9854, indicating high predictive performance. The most influential environmental variables limiting the distribution of *B. tsuneonis* were the Precipitation of Warmest Quarter (Bio18) and Temperature Seasonality (standard deviation ×100) (Bio4). Under current climatic conditions, potentially suitable habitat for *B. tsuneonis* in China covered an area of 215.9 × 10^4^ km^2^, accounting for 22.49% of the country’s land area. Potentially suitable habitat was primarily concentrated in Central China, South China, and East China. However, under future climatic projections, the area of suitable habitat for *B. tsuneonis* exhibited varying degrees of expansion. Furthermore, the centroid of the total suitable habitat for this pest gradually shifted westward and northward. These findings suggest that *B. tsuneonis* will spread to northern and western regions of China under future climate changes. The results of our study indicate that climate change will have a major effect on the invasion of *B. tsuneonis* and have implications for the development of strategies to control the spread of *B. tsuneonis* in China.

## 1. Introduction

*Bactrocera tsuneonis* (Miyake) (Diptera: Tephritidae) is a significant pest that exclusively infests citrus crops [1,2]. Previous economic loss assessments indicate that this pest typically leads to 10% to 20% reductions in citrus yields. However, if control measures are inadequate, the losses induced by this pest may exceed 50% [3]. Originally native to Japan, *B. tsuneonis* has been reported to occur in Sichuan [4], Guangxi [5], Guizhou [6], and Yunnan [7] Provinces in China. Larvae appear around the beginning of October, and each maggot devours between two to ten carpels. By early November, the mature larvae cause the infested fruit to drop to the ground, and they pupate in the top two inches of soil. Occasionally, larvae may pupate on the ground while the fruit remains on the tree [8].

The impact of *B. tsuneonis* on the citrus industry is progressively increasing due to climate change and increased international trade. This pest was first recorded in China in 1959 in the Ningming and Pingxiang regions of Guangxi Province. However, in 2016, *B. tsuneonis* was captured for the first time in Huaiji County, Guangdong Province, indicating that it could potentially spread to other regions [9]. Several studies have examined the adaptability of *B. tsuneonis* and methods to control its spread. Some approaches that have been examined include (1) fruit bagging [10], (2) the release of natural enemies [11], (3) traps [12], (4) the application of biopesticides [2], and (5) rapid molecular identification using microsatellite markers [13]. In a previous investigation by Wang et al. [14], the adaptability of *B. tsuneonis* in China was examined using CLIMEX and ArcGIS. Using climate data and data on the biological characteristics of the pest, they showed that *B. tsuneonis* is capable of adapting to 33 provinces in China, and the southern regions, which produce large amounts of citrus, were identified as highly suitable areas for this pest.

Knowledge of the potential distribution of invasive species is crucial for the development of effective policies and decision-making [15]. To this end, various algorithms have been developed for ecological niche modeling, which generally involve the use of presence and absence data in conjunction with environmental variables within a specific area. Some examples of these algorithms include MaxEnt, CLIMEX, GARP, and BIOCLIM [16,17,18,19,20,21]. The MaxEnt algorithm is widely used for modeling species distributions [16,22,23]. The MaxEnt algorithm estimates a target probability distribution by finding the distribution of maximum entropy, which approximates a uniform distribution, while adhering to a set of constraints that represent the incomplete information available about the target distribution [16]. This information typically includes a set of environmental variables, which are referred to as characteristics. Moreover, the expected constraints for each characteristic should correspond to the sample mean values obtained from the target distribution [16,24].

The MaxEnt model has been successfully used to predict the area of potentially suitable habitat for various invasive pests and economically significant insect pests, such as *Ceroplastes rusci* (Linnaeus, 1758) (Hemiptera: Coccidae), *Aleurodicus rugioperculatus* Martin, 2004 (Hemiptera: Aleyrodidae), *Riptortus pedestris* (Fabricius, 1775) (Hemiptera: Alydidae), *Daktulosphaira vitifoliae* (Fitch, 1855) (Hemiptera: Phylloxeridae), and *Spodoptera littoralis* (Boisduval, 1833) (Lepidoptera: Noctuidae) [25,26,27,28,29]. This model demonstrates high accuracy even when species distribution points are limited, which indicates that its predictive capability is superior to that of other models [30]. One common objective of these studies was to generate scenarios that could aid the planning and design of more efficient strategies for managing these pests at varying spatial scales. However, previous studies suggest that using default parameters in the MaxEnt model simulation can lead to overfitting, which reduces the transferability and accuracy of predictions [30]. To address this issue, Muscarella et al. [31] developed an R program package (ENMeval data package) to adjust the feature combination (FC) and regularization multiplier (RM) of the MaxEnt model. This can help identify model parameters with lower complexity and enhance prediction accuracy and stability [31]. For example, the FC and RM were optimized using the ENMeval data package in a study of *Linepithema humile* (Meyrick, 1868) (Lepidoptera: Tortricidae). Setting the FC to LQHPT (L = linear, Q = quadratic, H = hinge, P = product, and T = threshold) and the RM to 0.5 resulted in a highly accurate and stable model [32].

China ranks first among all countries in citrus planting area and second in citrus production. Citrus is an economically important fruit in the southern regions of China, and the prevalence of *B. tsuneonis* poses a major threat to citrus production in this region. The infestation rate of citrus fruits can reach 25% and even 100% under favorable environmental conditions [14]. Recognizing the severe threat posed by *B. tsuneonis* to China’s citrus industry, the Ministry of Agriculture included it in the national list of harmful quarantine organisms in 2009 [33]. Hence, there is a pressing need to predict the effect of climatic conditions on the potential distribution of *B. tsuneonis* in China. In this study, we developed an optimized MaxEnt model using *B. tsuneonis* distribution data, key environmental variables that limit its distribution, and the ENMeval data package. This model was used to predict the distribution of potentially suitable habitat for *B. tsuneonis* in China for different periods, including current and future periods (2021–2040, 2041–2060, 2061–2080, and 2081–2100), and under various future scenarios (SSP1-2.6, SSP2-4.5, SSP3-7.0, and SSP5-8.5). The aim of this study was to provide important information for citrus growers, local organizations, and governments that could facilitate the development of strategies to control the spread of *B. tsuneonis* in China.

## 2. Materials and Methods

### 2.1. Collection and Screening of Species Occurrence Data

**Figure 1:**
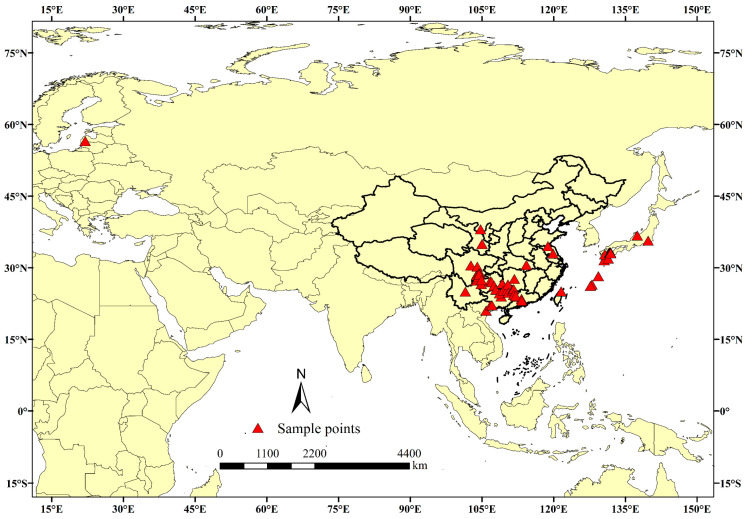
Global distribution of *B. tsuneonis* occurrence points.

A total of 85 occurrence records of *B. tsuneonis* (Figure 1) were obtained from various sources, including the GBIF Biodiversity Database (http://www.gbif.org/, accessed on 23 September 2023), the “National Directory of Agricultural Plant Quarantine Harmful Organisms Distribution by Administrative Region” (https://www.moa.gov.cn/nybgb/, accessed on 23 September 2023), Bold Systems v4 (http://www.boldsystems.org/, accessed on 25 September 2023), and the CABI International Centre for Applied Biological Sciences PlantwisePlus (https://plantwiseplusknowledgebank.org/, accessed on 28 September 2023), as well as previously published sources. The geographic coordinates for each distribution site were extracted from the literature or obtained using Google Earth Pro v7.3.4 (https://earth.google.com/web/, accessed on 28 September 2023). It is important to note that distribution site data are often biased toward easily accessible regions for humans or areas close to cities and other human settlements [34,35]. This bias can introduce spatial autocorrelation, which can significantly affect model results [36,37]. To address this issue and reduce sample bias, ENMTools 1.4 [38] was used to remove duplicate occurrences, which resulted in one distribution point per grid cell with a spatial resolution of 2.5 arc-minutes (approximately 4.5 km). After filtering, 69 occurrence points remained for model construction. For a detailed list of distribution points and corresponding maps, refer to Figure S1 in the Supplementary Materials. The workflow was implemented using ArcGIS 10.4 (ESRI, Redlands, CA, USA) (http://www.esri.com/, accessed on 3 October 2023).

### 2.2. Collection and Screening of Bioclimatic Variables

A total of 19 bioclimatic variables were obtained from the World Climate Database (https://www.worldclim.org/) at a resolution of 2.5 arc-minutes for both current and projected future climatic scenarios (Table 1). The bioclimatic variables were assessed using four shared socioeconomic pathways (SSP1-2.6, SSP2-4.5, SSP3-7.0, and SSP5-8.5), covering the current periods (1970–2000) and four future periods, including the 2030s (average for 2021–2040), 2050s (average for 2041–2060), 2070s (average for 2061–2080), and 2090s (average for 2081–2100). These scenarios were developed using the BCC-CSM2-MR global climate model from the National Climate Center. Each SSP represents a different level of radiative forcing: SSP1-2.6 corresponds to a low-forcing scenario, SSP2-4.5 corresponds to a medium-forcing scenario, SSP3-7.0 corresponds to a medium-to-high-forcing scenario, and SSP5-8.5 corresponds to a high-forcing scenario. The SSP1-2.6, SSP2-4.5, SSP3-7.0, and SSP5-8.5 scenarios assume that radiative forcing will stabilize at approximately 2.6, 4.5, 7.0, and 8.5 W/m^2^ by the year 2100, respectively.

**Figure 2:**
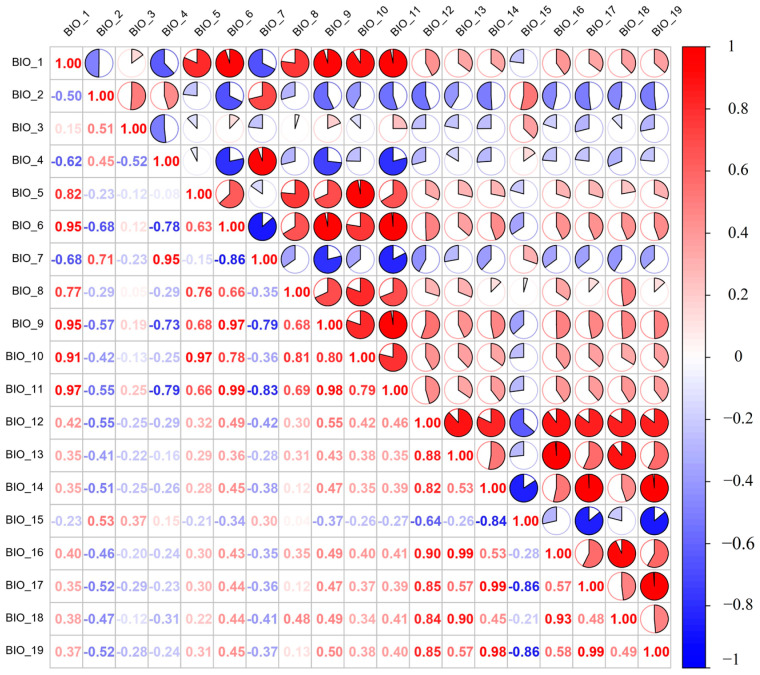
Pearson analysis plots for 19 environment variables (red indicates positive correlations, and blue indicates negative correlations).

To ensure the accuracy of our predictions and prevent potential correlations among climatic variables, we utilized the procedure outlined by Cai et al. [39]. First, the occurrence points of *B. tsuneonis* and the 19 bioclimatic variables were imported into MaxEnt software to create an initial model, with the random test percentage set to 25%. Subsequently, a jackknife test was performed to assess the percent contribution and permutation contribution of each variable to the initial simulation results. Next, to remove spatial autocorrelation among variables, the collected distribution data were used to extract values for 19 environmental variable layers in ArcGIS 10.4.1; the extracted values were then used to perform Pearson correlation analysis on the filtered variables using R software (Figure 2). Variables with correlation coefficients greater than |0.8| (indicating high correlations) were removed. From each pair of highly correlated variables, one was retained based on the percent contribution and permutation importance for modeling the potential distribution of *B. tsuneonis* [40], which facilitated the identification of the main environmental factors for modeling. Six bioclimatic variables were used in the final MaxEnt model (Table 1).

### 2.3. Model Optimization

In this study, the ENMeval package in R 4.3.2 was used to optimize the Maxent model [41]. The block method was used to partition the 69 *B. tsuneonis* records into four approximately equal parts, with three parts used for training and one part used for testing [31]. The RM parameter was set from 0.5 to 4 with an interval of 0.5; there was thus a total of 8 RM parameters [36,42]. For the FC parameters, the Maxent model had five features, linear (L), quadratic (Q), hinge (H), product (P), and threshold (T), from which eight feature combinations were derived (L, LQ, LQP, QHP, LQH, LQHP, QHPT, and LQHPT) [16]. A total of 64 parameter combinations were selected and tested using the ENMeval package. The model’s fit and complexity were assessed using the Akaike information criterion correction (AICc) [43,44], and the extent of overfitting was evaluated using a 10% training omission rate (OR_10_) [18,45]. The parameter combination with the smallest delta.AICc value was used in the final model.

### 2.4. Model Evaluation and Distribution of Potentially Suitable Habitat

The 69 *B. tsuneonis* occurrence records and six bioclimatic variables were input into Maxent 3.4.1 (Maxent (amnh.org, accessed on 12 December 2023)). The FC and RM were established according to the optimal model.c For simulation training, 75% of the occurrence records were selected, and the remaining 25% were used for model testing. In the MaxEnt model, the maximum number of iterations was set to 5000, with 10,000 as the maximum number of background points, and the logistic output format was used. The model was cross-validated by running 10 replicates. The jackknife method was used to test and create response curves, which was used to assess the effects of bioclimatic variables on the area of potentially suitable habitat of *B. tsuneonis* in China, and the accuracy of the model was examined using the area enclosed by the receiver operating characteristic (ROC) curve (AUC) [46]. The model prediction accuracy was categorized as excellent for AUC values between 0.9 and 1, good for values between 0.8 and 0.9, usable for values between 0.7 and 0.8, poor for values between 0.6 and 0.7, and failure for values between 0 and 0.5 [47].

In this study, the final results comprised average values from 10 repetitions in the MaxEnt model. The area of potentially suitable habitat for *B. tsuneonis* in China was delineated using a map of China. The results were obtained by assessing the presence probability of *B. tsuneonis*, with values ranging from 0 to 1, where higher values indicated a greater likelihood of species presence. The reclassify tool in ArcGIS software was used to categorize habitats with different levels of suitability using the natural breaks (Jenks) method. This resulted in the classification of areas into four levels: ‘highly suitable area’ (0.54 ≤ probability of occurrence ≤ 1), ‘moderately suitable area’ (0.33 ≤ probability of occurrence < 0.54), ‘marginally suitable area’ (0.11 ≤ probability of occurrence < 0.33), and ‘unsuitable area’ (0 ≤ probability of occurrence < 0.11).

The centroid is a useful measure for describing the spatial distribution of geographical objects and can also be used to track the displacement of these objects over time. In this study, we investigated the centroid shifts of *B. tsuneonis* within nationally suitable habitats under projected future climatic conditions. To do this, we first converted the habitat raster map into a vector map using ArcGIS software. Next, we analyzed it by inputting the folder containing current and future binary SDMs (species distribution models) into the SDMtoolboxw2.4 tool [48].

## 3. Results

### 3.1. Model Evaluation and Area of Potentially Suitable Habitat

The default parameters of the Maxent model were RM = 1 and FC = LQPHT. The ENMeval package was used to optimize the Maxent parameter settings. Figure S2 demonstrates that the model’s AICc value was the lowest (Delta.AICc = 0) when RM = 1.5 and FC = LQH. Model complexity was the lowest for this particular parameter combination based on the Akaike information criterion. The mean OR_10_ value was 30.64% lower with this particular parameter combination compared with the default parameter combination. The degree of overfitting was lowest with these parameter settings; thus, RM = 1.5 and FC = LQH were considered the optimal model parameters.

The performance of the MaxEnt model for *B. tsuneonis* outperformed the model under default settings, with an average test AUC value of 0.985 ± 0.007 (Figure S3). When the optimal parameter settings were used, the mean AUC values of the *B. tsuneonis* MaxEnt model exceeded 0.98 under different climate scenarios, which indicated that the model had high prediction accuracy and stability (Table 2).

### 3.2. Evaluation of Important Bioclimatic Variables

Table 3 shows the percentage contribution and permutation importance values for the six bioclimatic variables. Precipitation of Warmest Quarter (Bio18) was the most important bioclimatic variable, and its contribution rate and permutation importance were 67.5% and 7.9%, respectively. This suggested that Bio18 was the primary determinant of rainfall, which affected the distribution of *B. tsuneonis*. Additionally, the percent contribution of Temperature Seasonality (standard deviation ×100) (Bio4) and Mean Diurnal Temperature Range (Bio2) was 20.5% and 6.1%, respectively.

**Figure 3:**
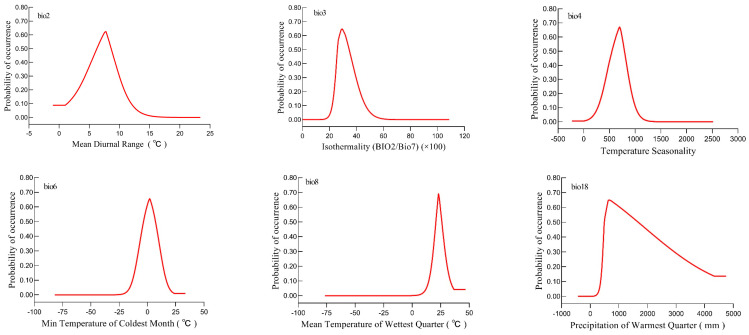
Probability of occurrence of *B. tsuneonis* for different values of the important bioclimatic variables.

The relationship between the presence probability of *B. tsuneonis* and bioclimatic variables is shown in Figure 3. Within a specific range, the probability of the occurrence of *B. tsuneonis* increased as Bio2, Bio3, Bio4, Bio6, Bio8, and Bio18 increased. After peaking, the probability of occurrence of *B. tsuneonis* decreased with further increases in environmental factors. The average ranges of suitable values for these bioclimatic variables (probability ≥ 0.33) were as follows: 4.61–9.76 °C for Bio2, 24.67–38.93 for Bio3, 441.60–867.63 for Bio4, −7.23–10.81 °C for Bio6, 18.87–28.38 °C for Bio8, and 450.22–2735.93 mm for Bio18.

### 3.3. Potentially Suitable Habitat for B. tsuneonis under Current Climate Conditions

**Figure 4:**
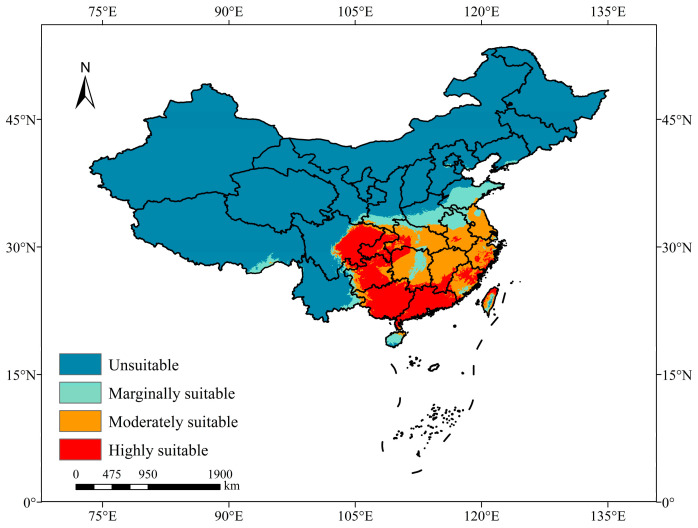
Potentially suitable areas for *B. tsuneonis* under current climatic conditions in China.

The current potential distribution map for *B. tsuneonis* in China is shown in Figure 4. The suitable areas for *B. tsuneonis* were primarily located in central China, East China, South China, and the eastern region of Southwest China. Under current climate conditions, the area of potentially suitable habitat for *B. tsuneonis* in China was approximately 215.9 × 10^4^ km^2^, which accounted for 22.49% of China’s area.

The highly suitable, moderately suitable, and marginally suitable areas comprised 82.6 × 10^4^ km^2^, 82.05 × 10^4^ km^2^, and 51.26 × 10^4^ km^2^, which accounted for 8.60%, 8.55%, and 5.34% of the total area of China, respectively. These areas were primarily distributed in southern regions below 40 °N, including Chongqing, Guizhou, Guangxi, Hubei, Hunan, Guangdong, Jiangxi, Fujian, Anhui, Zhejiang, Jiangsu, Shandong, Hainan, and Taiwan, as well as parts of Yunnan, Gansu, Shaanxi, and Henan. Highly suitable areas were particularly prevalent in various parts of Guangzhou, Guangxi, Guizhou, Chongqing, and eastern Sichuan.

### 3.4. Changes in the Area of Potentially Suitable Habitat for B. tsuneonis under Future Climatic Scenarios

**Figure 5:**
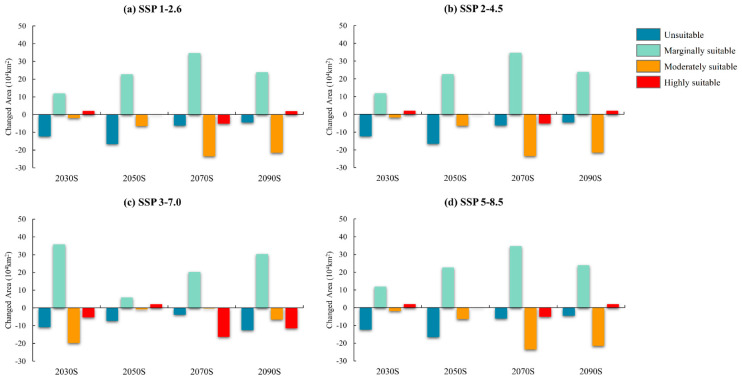
Differences in the area of suitable habitat in future and current periods (future–current) under different climate change scenarios in China.

**Figure 6:**
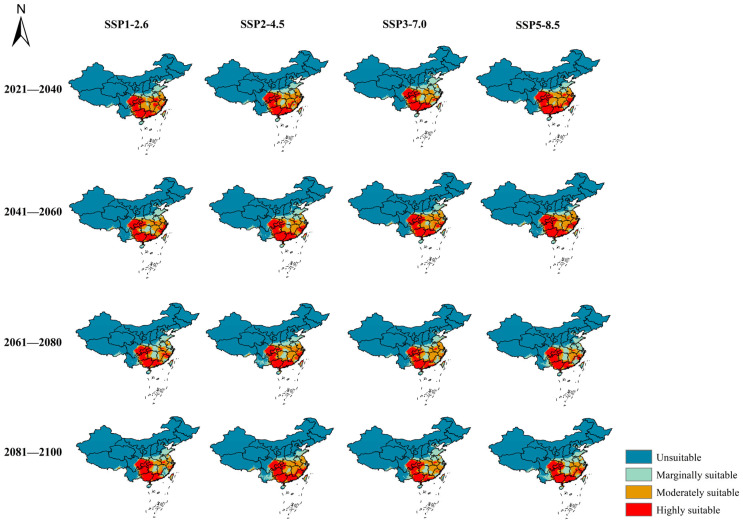
Potential habitat suitability of *B. tsuneonis* under different climate change scenarios in China.

The potential distribution of *B. tsuneonis* based on four emission scenarios (SSP1-2.6, SSP2-4.5, SSP3-7.0, and SSP5-8.5) across four future periods (2030s, 2050s, 2070s, and 2090s) is shown in Figure 5 and Figure 6, and Table 4. The total suitable area for *B. tsuneonis* was projected to increase to varying degrees under future climate scenarios compared with current climate conditions. The largest areas of suitable habitat were observed in Chongqing, Guizhou, Guangxi, and Guangdong.

In conclusion, the area of potentially suitable habitat increased in the western and northern regions of China in the future periods under the four emission scenarios. Under the SSP5-8.5 emission scenario, the total suitable area reached its maximum value of 237.77 × 10^4^ km^2^ in the 2090s, and the area of highly suitable habitat peaked at 92.78 × 10^4^ km^2^ in the 2030s.

### 3.5. Centroid Shifts of Potentially Suitable Areas for B. tsuneonis

**Figure 7:**
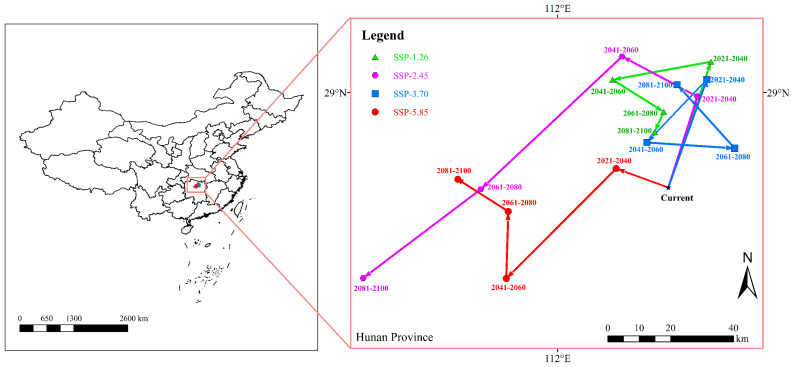
Changes in geographical centers of the area of potentially suitable habitat for *B. tsuneonis* under current and future climatic scenarios.

The centroids of potentially suitable areas of *B. tsuneonis* in China under current and future climate scenarios are shown in Figure 7 and Table 5. Currently, the species’ range centroid is located in Yiyang City, Hunan Province (28.727 °N, 112.317 °E). The centroid of potentially suitable areas for *B. tsuneonis* is predicted to shift westward and northward under future climate scenarios.

## 4. Discussion

### 4.1. Significance of the Optimal Model Predictions

Insects have caused considerable damage to crops for centuries [49], and they are generally responsible for the loss of approximately 40% of agricultural production [50]. Insect pests pose a major threat to agricultural production in China [51], especially commercial citrus production. Numerous climatic suitability studies have been conducted on various citrus insect pests in China including *Trioza erytreae* (Del Guercio, 1918) (Hemiptera: Triozidae) [52], *Diaphorina citri* (Kuwayama, 1908) (Hemiptera: Liviidae) [53,54], *Bactrocera dorsalis* (Hendel, 1912) (Diptera: Tephritidae) [55], and *Anoplophora chinensis* (Forster, 1771) (Coleoptera: Cerambycidae) [56].

Monitoring the distribution of pests is critically important for determining the areas where invasive species might potentially colonize as a result of global temperature increases, which could aid the development of strategies to control these pests [25,26]. We used an optimized MaxEnt model to predict the potential distribution of *B. tsuneonis* under climate change and identify the significant variables affecting the distribution of its potentially suitable habitat in China. Clarifying the future expansion of *B. tsuneonis* can aid the development of strategies to control their spread and mitigate the damage induced by this pest under future climate change. Therefore, our findings have implications for preventing the further spread of *B. tsuneonis* in China.

MaxEnt typically selects a random subset of data for data modeling and evaluates the model’s prediction ability using the AUC, but the software has certain limitations [57]. First, when both the training data and test data are affected by sampling deviation, the AUC might overestimate the model’s predictive accuracy [58]. Second, the complexity of the MaxEnt model, which is a complex machine learning algorithm, can lead to overfitting when simulating the potential distributions of species, which can affect the transferability of the model [23]. To address this, adjustments can be made to the RM and the FC using AICc [45]. We used the ENMeval package to optimize the predictive performance of the MaxEnt model by integrating multiple parameters, and this package has been shown to be more effective for model optimization compared with other packages [59]. When the optimal parameters of the MaxEnt model for predicting the suitable habitat for *B. tsuenoenis* were used (RM = 1.5, FC = LQH), the delta.AICc = 0, and the remodeled training AUC exceeded 0.98. This indicated that the predictive accuracy of the model was enhanced, which improved the ability of this model to clarify the relationships between environmental variables and occurrence data. Wang et al. [14] used CLIMEX to predict the distribution of suitable habitat for *B. tsuneonis* under current climatic conditions and found that the suitable habitat for *B. tsuneonis* in China was primarily concentrated in the East, Central, South, and Southwest regions. These findings are consistent with the results of this study and support the accuracy of our predictions.

### 4.2. The Bioclimatic Variables Determining the Distribution of Suitable Habitat for B. tsuneonis

Precipitation has direct and indirect effects on crop insect pests [60]. The results of this study confirmed that precipitation was a key environmental factor affecting the distribution of *B. tsuneonis*. The factor with the greatest effect on the distribution of *B. tsuneonis* was Bio18, which was precipitation during the warmest quarter, and the range of suitable values for this variable was 450.22–2735.93 mm. During its annual life cycle, the citrus fruit fly overwinters as mature larvae that enter the soil for pupation, and the larvae spend one stage in the soil. Precipitation directly affects soil moisture and can subsequently affect the emergence of overwintering pupae [61]. Precipitation also directly affects air humidity, as the citrus fruit fly tends to emerge after rainfall, and the optimal humidity range for the growth and development of adults ranges from 50% to 90% [14]. Moreover, precipitation affects the growth of host plants for the citrus fruit fly, which can have consequences for the growth and development of the flies. Previous studies have confirmed that annual rainfall between 1300 and 1500 mm is favorable for the growth of citrus [62], which coincides with the projected precipitation range required for the suitable habitat of *B. tsuneonis*. In Guizhou Province, a significant proportion of highly suitable habitat for *B. tsuneonis* was observed, and the average annual rainfall over the past 30 years has ranged from 900 to 1300 mm [63]. This is consistent with the precipitation range predicted to be suitable for *B. tsuneonis* in this study.

The results of this study confirmed that temperature was a key environmental variable affecting the distribution of *B. tsuneonis*. The results indicated that environmental variables associated with temperature included Bio2 (Mean Diurnal Temperature Range); Bio3 (Isothermality) (Bio2/Bio7) (×100); Bio4 (Temperature Seasonality) (standard deviation ×100); Bio6 (Minimum Temperature of Coldest Month); and Bio8 (Mean Temperature of Wettest Quarter). Numerous studies have indicated that temperature is a key climatic factor affecting the population dynamics of fruit fly pests in the field [64,65,66,67,68]. Ma et al. [69] conducted experiments in which larvae collected from infested fruits were reared indoors until emergence, and observations under a temperature gradient ranging from 0 to 24 °C revealed that no adults emerged at temperatures of 9 °C and below, indicating that the minimum developmental temperature for *B. tsuneonis* pupae is above 9 °C. Yasuda et al. [70] conducted temperature treatments on *B. tsuneonis* pupae and observed developmental arrest at 15 °C and 25 °C. These studies collectively demonstrate that temperature is one of the key environmental variables affecting the distribution of *B. tsuneonis*.

Moreover, *B. tsuneonis* is an oligophagous pest that primarily targets citrus fruits [71]. For example, Yongchun County in Quanzhou City, Fujian Province is known as the “Home of Chinese citrus.” The results of this study indicated that Yongchun County was highly suitable for the proliferation of *B. tsuneonis*, both under current conditions and under projected future climate scenarios. Over the past 20 years, the lowest recorded temperature in Yongchun County was −2.1 °C, and the highest temperature has ranged between 36.5 and 39 °C [72]. The anticipated temperature threshold for the suitability of *B. tsuneonis* in our study was consistent with the annual temperature range in this area, which confirms the robustness of our findings.

### 4.3. Prospective Changes in the Distribution of Suitable Habitat for B. tsuneonis

Currently, suitable habitat for *B. tsuenonis* in China under current climatic conditions is primarily located in provinces such as Chongqing, Guizhou, Guangxi, Hubei, Hunan, Guangdong, Jiangxi, Fujian, Anhui, Zhejiang, Jiangsu, Shandong, Hainan, and Taiwan. The habitats in these regions exhibit varying degrees of suitability, including high, medium, and marginal suitability. In some areas, high suitability areas cover more than 60% of the total suitable area. Ten of these regions are also known to have large citrus planting areas and are the top citrus-producing regions in China [73]. Therefore, caution is needed to prevent the spread of *B. tsuneonis* to these regions. Xia et al. [74] conducted a two-year monitoring study on *B. tsuneonis* in citrus orchards located in Pinghe County, Zhangzhou City, and Fujian Province. Although they did not observe *B. tsuneonis* during the study period, they emphasized the significance of not ignoring this pest given that Pinghe County is known for its extensive production of Guanxi honey pomelo, which serves as a preferred host for *B. tsuneonis*. Furthermore, our findings revealed that Pinghe County was highly suitable for the proliferation of *B. tsuneonis*, both under current conditions and under projected future climate scenarios. Therefore, the invasion of *B. tsuneonis* in this region would have a significant effect on the local citrus industry due to the abundance of food resources and favorable environmental conditions.

Numerous studies have indicated that climate change will modify the potential distribution of insect pests in a species-specific manner [75,76,77]. Climate change can result in the expansion of the potential distributions of certain insect pests [78] and cause contractions in the potential distributions of others [79]. Additionally, climate change can prompt species to migrate north or toward higher latitudes [80]. These effects are particularly evident in high-altitude regions, as temperature increases in these areas will be more pronounced than in lower altitudes [81]. The fate of insect pests is largely determined by their ability to adapt to rising temperatures and fluctuating rainfall.

Under future climate scenarios, the potential distribution of *B. tsuneonis* will gradually expand toward higher latitudes until the 2090s, which is likely attributed to the global warming trend. These findings are consistent with predicted changes in the potential distributions of other invasive pests, such as *Spodoptera frugiperda* [39], *Solenopsis invicta* [82], and *Culex pipiens pallens* [83]. The prediction results generated by the MaxEnt model suggest that regions such as Guizhou, Sichuan, Yunnan, Zhejiang, and Jiangxi will be affected under various climate change scenarios, indicating that there will be an increase in the total suitable habitat area for the citrus fruit fly. Moreover, our findings will aid the development of strategies to prevent the spread of this pest to areas such as Jiangxi, Hubei, Chongqing, and Fujian, where the citrus fruit fly has not yet been observed.

### 4.4. Limitations of This Research

We evaluated the area of suitable habitat for the citrus fruit fly; however, other factors aside from climate can also affect the fly’s distribution. Biotic interactions, including variables such as crop yields, natural enemies, pests, weeds, and plant diseases, play a significant role in determining the distributions of various insects [84,85]. Additionally, temperature and precipitation, which are the main factors affecting the abundance and distribution of species, not only affect the physiology of pests but also affect the physiology of host plants, which can subsequently affect the pests themselves [86]. Furthermore, it is important to note that studies of spatial distributions have inherent uncertainties that can be related to various factors, including future greenhouse gas emission levels, the extent of climate change projections, the parameterization of the model, and the availability of broad-scale climate data [87,88,89]. For example, climate change-induced increases in CO_2_ levels can increase the carbon/nitrogen ratio in plants, which leads to a decrease in the protein content. This can result in pests causing more damage as they compensate for reduced food quality [84].

Furthermore, our study did not account for the effects of evolutionary and adaptive processes that likely affect insects, including the citrus fruit fly [88,89]. Although projections of the effects of climate change on insects typically assume that species’ thermal requirements remain static and do not evolve, the physiological requirements of species can be flexible. Insects may respond differently to environmental pressures through processes such as acclimation and diapause quiescence [88]. Nonetheless, this finding indicates that models, such as MaxEnt, provide predictions that are consistent with the realized niche, which represents the actual environment inhabited by the species [90]. The resulting maps are indicators of the potential future invasion of *B. tsuneonis*; additional research on this economically significant agricultural pest and its socioeconomic impact is urgently needed.

## 5. Conclusions

Our study is the first to utilize an optimized MaxEnt model to investigate the distribution of suitable habitat for *B. tsuneonis* in China and the key bioclimatic variables determining the habitat suitability of *B. tsuneonis*. Our findings reveal that the area of suitable habitat for this fruit fly pest is projected to increase and shift toward higher latitudes under future climate scenarios relative to that under current climate conditions. Precipitation of Warmest Quarter (Bio18) and Temperature Seasonality (standard deviation ×100) (Bio4) were the key factors determining the distribution of this pest. These findings emphasize the major role of climate change in affecting the potential distribution of *B. tsuneonis*. Generally, the establishment of a network for monitoring this pest is essential for preventing its future spread in citrus-planting areas throughout China.

## Figures and Tables

**Table 1:** Bioclimatic variables used to model the potentially suitable habitat of *B. tsuneonis* in China. The six bioclimatic variables used for model development are indicated in bold.

Variables	Description	Unit
Bio1	Annual Mean Temperature	°C
**Bio2**	**Mean Diurnal Temperature Range**	**°C**
**Bio3**	**Isothermality (Bio2/Bio7) (×100)**	**/**
**Bio4**	**Temperature Seasonality (standard deviation ×100)**	**/**
Bio5	Maximum Temperature of Warmest Month	°C
**Bio6**	**Minimum Temperature of Coldest Month**	**°C**
Bio7	Temperature Annual Range (Bio5-Bio6)	°C
**Bio8**	**Mean Temperature of Wettest Quarter**	**°C**
Bio9	Mean Temperature of Driest Quarter	°C
Bio10	Mean Temperature of Warmest Quarter	°C
Bio11	Mean Temperature of Coldest Quarter	°C
Bio12	Annual Precipitation	mm
Bio13	Precipitation of Wettest Month	mm
Bio14	Precipitation of Driest Month	mm
Bio15	Precipitation Seasonality (Coefficient of Variation)	/
Bio16	Precipitation of Wettest Quarter	mm
Bio17	Precipitation of Driest Quarter	mm
**Bio18**	**Precipitation of Warmest Quarter**	**mm**
Bio19	Precipitation of Coldest Quarter	mm

**Table 2:** Mean AUC values of the MaxEnt model for *B. tsuneonis* across different climate scenarios.

Climate Scenario	Year	AUC Value
-	Current	0.985
Lowly compulsive scenario SSP1-2.6	2021–2040	0.988
	2041–2060	0.986
	2061–2080	0.981
	2081–2100	0.992
Moderately compulsive scenario SSP2-4.5	2021–2040	0.986
	2041–2060	0.990
	2061–2080	0.985
	2081–2100	0.989
Moderately to highly compulsive scenario SSP3-7.0	2021–2040	0.981
	2041–2060	0.989
	2061–2080	0.985
	2081–2100	0.984
Highly compulsive scenario SSP5-8.5	2021–2040	0.987
	2041–2060	0.989
	2061–2080	0.986
	2081–2100	0.987

**Table 3:** Percent contribution and permutation importance of the six main bioclimatic variables.

Variables	Percent Contribution (%)	Permutation Importance (%)
Bio18	67.5	7.9
Bio4	20.5	21.5
Bio2	6.1	0.2
Bio6	2.4	0.2
Bio8	2.3	36.6
Bio3	1.3	33.7

**Table 4:** Changes in the area of suitable habitats for *B. tsuneonis* under different climate scenarios.

Scenario	Decade	Total Suitable Regions		Regions of Marginally Suitable Habitat		Regions of Moderately Suitable Habitat		Regions of Highly Suitable Habitat	
		Area (×10^4^ km^2^)	Area Change (%)	Area (×10^4^ km^2^)	Area Change (%)	Area (×10^4^ km^2^)	Area Change (%)	Area (×10^4^ km^2^)	Area Change (%)
-	Current	215.90	-	51.26	-	82.05	-	82.60	-
SSP1-2.6	2030s	228.15	5.67%	63.26	23.43%	80.22	−2.23%	84.67	2.51%
	2050s	232.42	7.65%	74.03	44.44%	75.64	−7.81%	82.75	0.19%
	2070s	222.05	2.85%	85.97	67.72%	58.59	−28.59%	77.50	−6.17%
	2090s	220.35	2.06%	75.18	46.68%	60.55	−26.21%	84.62	2.46%
SSP2-4.5	2030s	225.75	4.56%	63.41	23.71%	78.12	−4.79%	84.22	1.97%
	2050s	236.30	9.45%	75.29	46.89%	71.28	−13.13%	89.73	8.64%
	2070s	236.36	9.47%	74.17	44.70%	81.82	−0.29%	80.38	−2.69%
	2090s	233.31	8.06%	62.32	21.58%	79.55	−3.05%	91.44	10.71%
SSP3-7.0	2030s	226.64	4.97%	87.05	69.84%	62.30	−24.08%	77.29	−6.42%
	2050s	223.19	3.38%	57.23	11.66%	81.30	−0.92%	84.67	2.51%
	2070s	219.60	1.71%	71.63	39.74%	81.72	−0.40%	66.25	−19.79%
	2090s	228.38	5.78%	81.73	59.46%	75.55	−7.93%	71.11	−13.91%
SSP5-8.5	2030s	221.71	2.69%	54.25	5.84%	74.68	−8.98%	92.78	12.33%
	2050s	220.94	2.33%	50.71	−1.06%	79.11	−3.58%	91.12	10.32%
	2070s	226.93	5.11%	92.18	79.84%	55.92	−31.84%	78.83	−4.56%
	2090s	237.77	10.13%	92.41	80.30%	59.24	−27.81%	86.12	4.27%

**Table 5:** Changes in the geographical center of the area of potentially suitable habitat for *B. tsuneonis* under current and future climatic scenarios.

Current Centroid Location	Climate Scenario	Future Centroid Location			
		2030s	2050s	2070s	2090s
Yiyang City, Hunan Province (112.317 °E, 28.727 °N)	SSP1-2.6	Yiyang City (29.087 °N, 112.439 °E)	Changde City (29.034 °N, 112.157 °E)	Yiyang City (28.942 °N, 112.303 °E)	Yiyang City (28.884 °N, 112.277 °E)
	SSP2-4.5	Yiyang City (28.988 °N, 112.400 °E)	Changde City (29.104 °N, 112.184 °E)	Changde City (28.723 °N, 111.780 °E)	Yiyang City (28.468 °N, 111.443 °E)
	SSP3-7.0	Yiyang City (29.038 °N, 112.426 °E)	Changde City (28.858 °N, 112.253 °E)	Yiyang City (28.840 °N, 112.506 °E)	Yiyang City (29.024 °N, 112.340 °E)
	SSP5-8.5	Changde City (28.783 °N, 112.167 °E)	Yiyang City (28.468 °N, 111.852 °E)	Changde City (28.660 °N, 111.858 °E)	Changde City (28.752 °N, 111.714 °E)

## Data Availability

The raw data supporting the conclusions of this article will be made available by the authors on request.

## Supplementary Materials

The following supporting information can be downloaded at: https://www.mdpi.com/article/10.3390/insects15060417/s1. Figure S1: Occurrence data of *B. tsuneonis* for MaxEnt modeling. Figure S2: Evaluation results of the MaxEnt model under different settings. (a) Delta.AICc; (b) OR_10_. Legends denote different feature classes (L = linear, Q = quadratic, H = hinge, P = product, and T = threshold). Figure S3: Receiver operating characteristic (ROC) curve of the MaxEnt model. The plot represents the sensitivity (true positive rate) and the specificity (false positive rate) of the model. The area under the ROC curve (AUC) represents the entire area underneath the ROC curve (red); the 95% confidence intervals are indicated in blue.
