# Supplementary material for: Forecasting the Expansion of Bactrocera tsuneonis (Miyake) (Diptera: Tephritidae) in China Using the MaxEnt Model
Source: Insects. 2024 Jun 4;15(6):417. doi: 10.3390/insects15060417 (PMC11203975; doi:10.3390/insects15060417)
Supplement: Supplementary file 1 [file insects-15-00417-s001.zip › insects-3001228-supplementary.pdf]

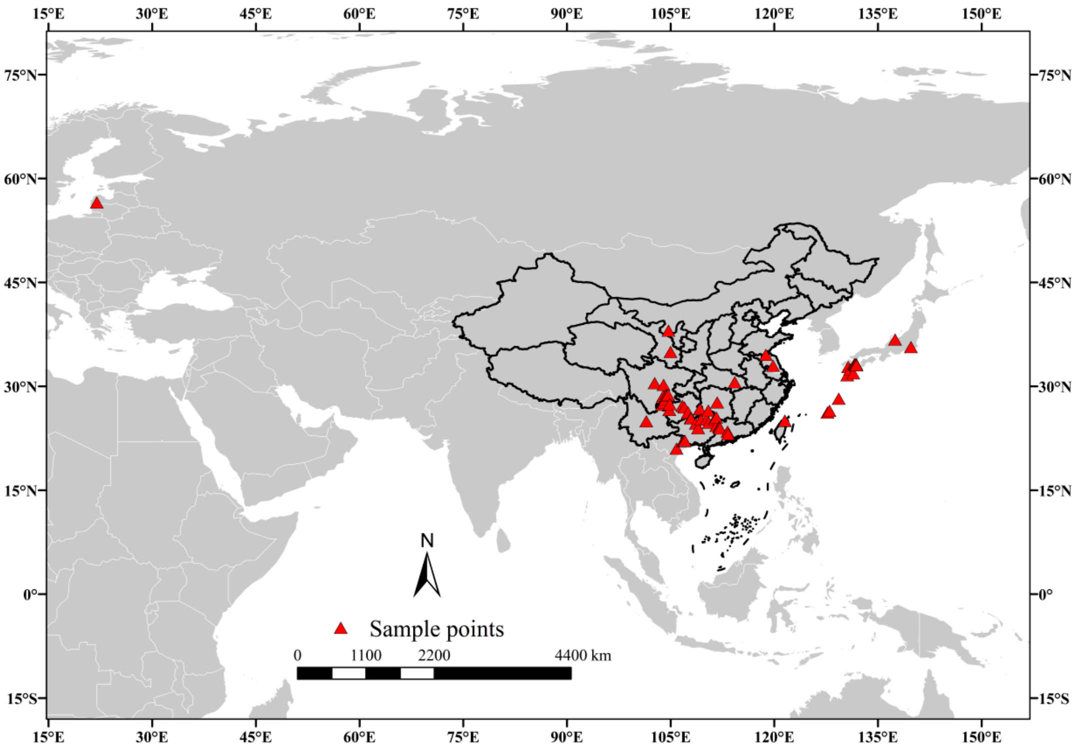

Figure S1: Occurrence data of *B. tsuneonis* for the MaxEnt modeling.

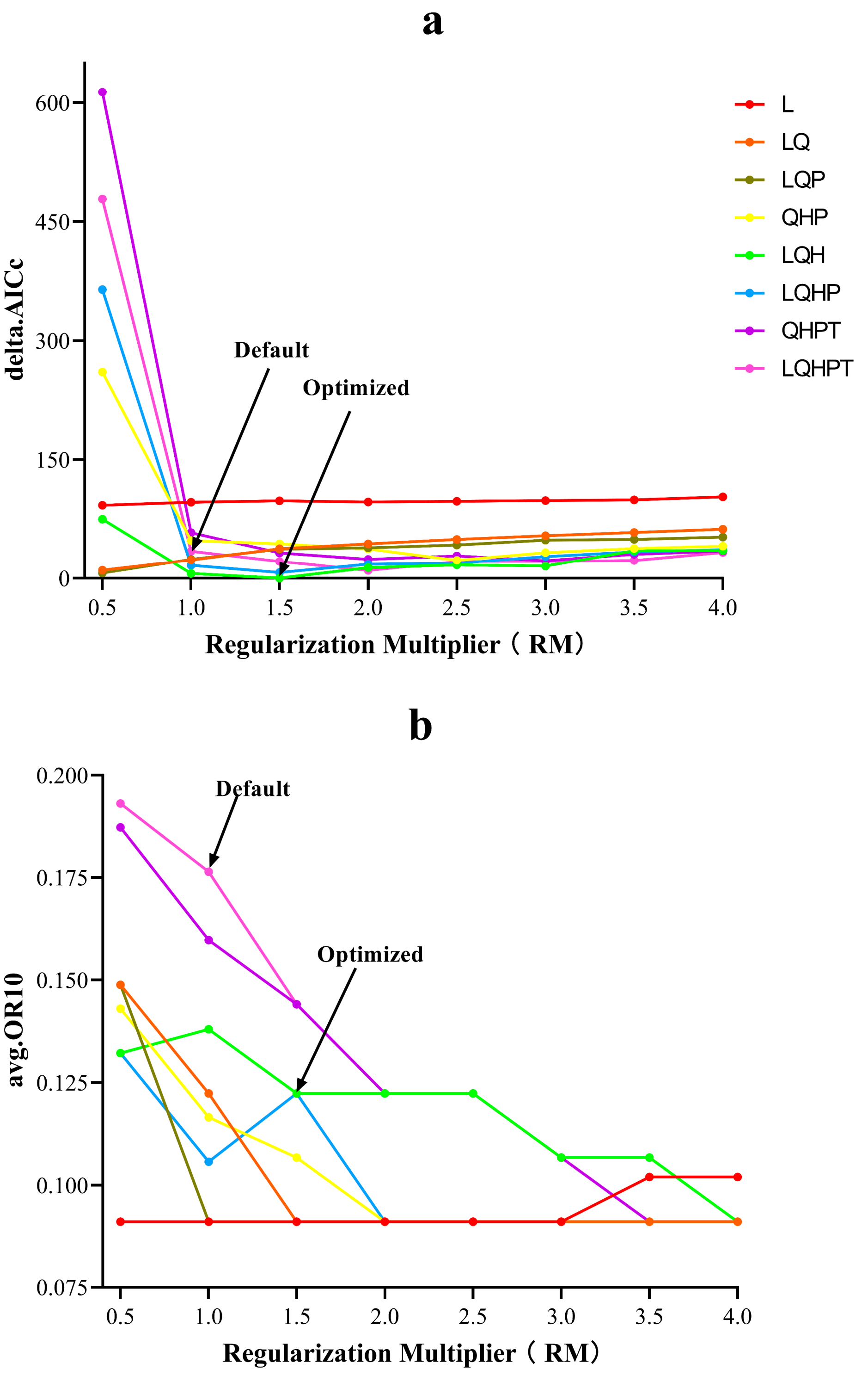

Figure S2: Evaluation results of the MaxEnt model under different settings.(a) Delta.AICc; (b) OR_{10}. Legends denote different feature classes (L = linear, Q = quadratic, H = hinge, P = product and T = threshold).

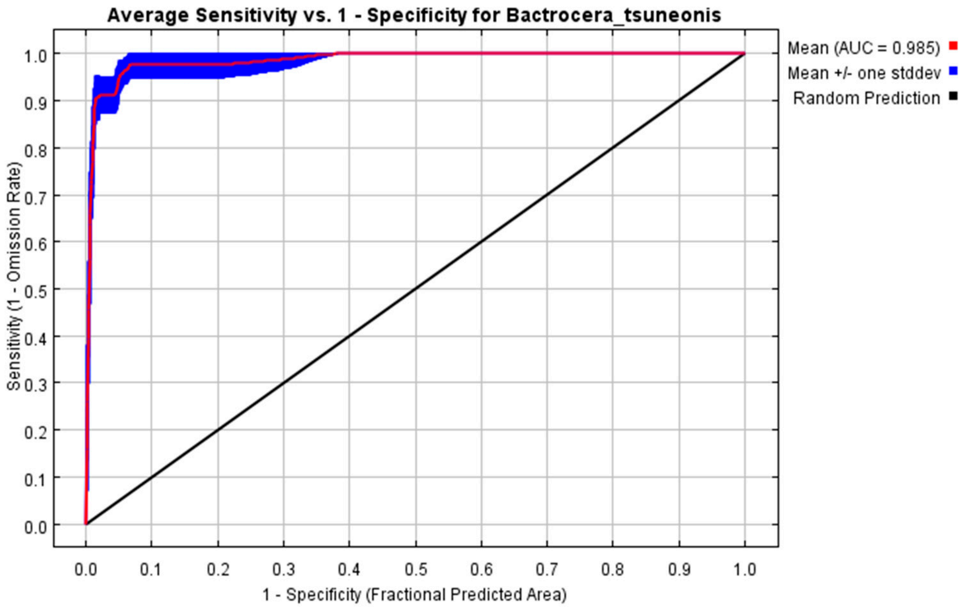

Figure S3: Receiver operating characteristic (ROC) curve of the MaxEnt model. The plot represents the sensitivity (true positive rate) and the specificity (false positive rate) of the model. The area under the ROC curve (AUC) represents the entire area underneath the ROC curve (red); the 95% confidence intervals are indicated in blue.
